# Papel do Ômega-3 e do Ômega-6 sobre Fatores de Risco Cardiovasculares: Importância da Fonte da Dieta e da Estrutura do Lipídio

**DOI:** 10.36660/abc.20230753

**Published:** 2023-12-07

**Authors:** Victória Moralez Soares, Thais Caroline da Silva, Priscila Portugal dos Santos

**Affiliations:** 1 Universidade Estadual Paulista Júlio de Mesquita Filho Faculdade de Medicina de Botucatu Botucatu SP Brasil Universidade Estadual Paulista Júlio de Mesquita Filho – Campus de Botucatu – Faculdade de Medicina de Botucatu , Botucatu , SP – Brasil

**Keywords:** Dieta do Mediterrâneo, Gorduras na Dieta, Fatores de Risco, Ácidos Graxos Omega-3, Ácidos Graxos Omega-6, Receptores, Eicosanoides

Ômega-3 (ácido α-linolênico - ALA, C18:3n-3) e ômega-6 (ácido linoleico - LA, C18:2n-6), são ácidos graxos poliinsaturados (AGPI) essenciais, pois não podem ser sintetizados por humanos ou outros animais superiores. ^1^ Estes AGPI desempenham um papel importante na manutenção da saúde cardiovascular, sendo extensivamente estudados. A dieta mediterrânica tem sido repetidamente associada a menor risco cardiovascular, em grande parte devido à abundância de ómega-3. ^2^

No corpo humano, ALA e LA são convertidos em outras formas de AGPI, importantes para a saúde humana, incluindo ácido eicosapentaenoico (EPA, C20:5n-3), ácido docosapentaenoico (DPA, C22:5n-3), ácido docosaexaenoico (DHA, C22:6n-3) da família ômega-3, e ácido γ-linolênico (GLA, 18:3n-6), ácido dihomo-γ-linolênico (DGLA, 20:3n-6) e ácido araquidônico (AA, C20: 4n-6) da família ômega-6. ^1^

Geralmente considera-se que os AGPI desempenham um papel benéfico para a saúde. ^1^ No entanto, podem ter efeitos opostos, sendo o ómega-3 associado à redução do risco cardiovascular e o ómega-6 associado a fatores de risco cardiovascular, ^1 , 3^ como mostra o estudo de Gonçalinho et al., ^4^ Os efeitos dos AGPI no corpo e no sistema cardiovascular são devidos à geração de lipídios sinalizadores bioativos (derivados de DGLA, AA, EPA, DHA) conhecidos como eicosanoides. Entre os eicosanoides produzidos estão as prostaglandinas (PGs), tromboxanos (TXs) e leucotrienos (LTs). O ômega-3 também pode ser metabolizado em mediadores lipídicos especializados de pró-resolução, como resolvinas da série D, protetinas e maresinas. ^1 , 3 , 5^

Os eicosanoides derivados do ômega-6 estão envolvidos principalmente na ativação da resposta pró-inflamatória, agregação plaquetária, trombose e tônus vascular, o que pode levar à doença cardiovascular (DCV) aguda. ^2 , 5 , 6^ No entanto, alguns estudos sobre os efeitos do ômega-6 ainda são controversos, como discutido por Gonçalinho et al. ^4^ Alguns eicosanoides derivados de AA têm efeitos opostos. Por exemplo, o TXA2 promove vasoconstrição e agregação plaquetária levando à formação de trombos. PGI2 é um potente vasodilatador e inibe a agregação plaquetária. Assim, o equilíbrio na produção desses eicosanoides estabelece o tônus vascular e o potencial trombótico. ^5^ Além disso, os eicosanoides derivados de DGLA, como PGE1, possuem principalmente propriedades anti-inflamatórias e inibem a agregação plaquetária. ^3^

Adicionalmente, os metabólitos derivados de AA também podem desempenhar um papel na resolução da inflamação. Por exemplo, a lipoxina A4 é um potente mediador pró-resolução ^7^ e a geração de eicosanoides derivados de AA no início da resposta inflamatória está associada à posterior indução da resolução da inflamação. ^6^ Essas considerações podem explicar os resultados controversos encontrados em estudos com ômega-6.

Por outro lado, os eicosanoides derivados do ômega-3 possuem ações anti-inflamatórias, vasodilatadoras e de antiagregação plaquetária, reduzindo assim o risco de DCV, ^1 , 2 , 5 , 8^ conforme demonstrado por Gonçalinho et al., ^4^ Quanto aos efeitos anti-inflamatórios, o EPA e o DHA diminuem a produção de eicosanoides derivados do AA, além de diminuir a ativação de fatores de transcrição pró-inflamatórios, levando a menor produção de citocinas e quimiocinas inflamatórias, proteínas de fase aguda e moléculas de adesão. ^5^ Além disso, o EPA e o DHA possuem efeitos de antiagregação plaquetária por modificar os perfis dos eicosanoides. ^9^

Outras ações interessantes do ômega-3 são regular o metabolismo lipídico e dos adipócitos e os níveis de colesterol, potencializando assim seu efeito na saúde cardiovascular. ^1 , 10 , 11^ O EPA e o DHA modificam beneficamente os lipídios sanguíneos e têm efeito hipotriglicêmico regulando vias de síntese e degradação lipídica. ^5^ Ele também atua na supressão das enzimas da biossíntese do colesterol. ^10^ Além disso, o ômega-3 atua no tecido adiposo marrom regulando o gasto energético, por meio de sua função termogênica. ^11^

Finalmente, o ômega-3 pode influenciar o efeito dos ácidos graxos saturados (SFA) e do ômega-6 no perfil lipídico. O SFA aumentam os níveis plasmáticos de triacilglicerol (TG) apenas quando a dieta é deficiente em ômega-3. Além disso, em indivíduos com índice adequado de ômega-3, a dieta SFA não afeta significativamente a concentração de partículas LDL ou os níveis de colesterol LDL. ^12^

Considerando os efeitos opostos do ômega-3 e ômega-6, a proporção desses AGPI desempenham papel significativo na regulação da homeostase corporal. A taxa de conversão de LA e ALA em outros metabólitos depende da proporção de ômega-3 e ômega-6 ingeridos, uma vez que eles competem pelas mesmas enzimas neste processo, embora exista uma afinidade preferencial destas enzimas pelo ômega-3. ^1 , 8^ A proporção dietética recomendada de ômega-6/ômega-3 para benefícios à saúde é de 1:1–2:1. No entanto, nas dietas ocidentais típicas, a proporção é de 15:1 a 16,7:1. ^1 , 13^

É também importante prestar atenção à qualidade da fonte destes AGPI, uma vez que a estrutura dos lípidos dietéticos e as posições de ligação dos ácidos graxos (AG) nestes lípidos podem influenciar a sua digestão, transporte e consequentemente a sua biodisponibilidade, metabolismo e efeitos sobre o coração. ^14 - 17^

Em alimentos de origem vegetal e animal a maioria (~98%) do ômega-3 é encontrada na forma de TG, seguido por fosfolipídios (PLs) e diacilgliceróis, ésteres de colesterol e ésteres vitamínicos solúveis em gordura. Estas diferentes formas de lipídios apresentam diferentes níveis de biodisponibilidade. ^14^ Os PLs são mais biodisponíveis devido à sua natureza anfifílica, com superior dispersibilidade em água e maior suscetibilidade às fosfolipases em comparação com a glicerólise do TG. ^14^ Estudos mostraram que uma dose mais baixa de óleo de Krill, (62,8% em comparação com o óleo de peixe) que contém quase 35% de DHA na forma de PL, foi mais eficiente do que o óleo de peixe em promover absorção do EPA e DHA, uma vez que, os AGPI no óleo de peixe são encontrados na forma de TG. Além disso, óleo de Krill causou menos efeitos adversos. ^15 , 18^ Outro estudo mostrou que bebês alimentados com fórmula contendo AGPI na forma de PL de ovo absorveram ômega-3 tão eficientemente quanto bebês amamentados, e melhor do que bebês alimentados com fórmula contendo AGPI na forma de TG. ^19^

Em relação ao transporte desses lipídios, no enterócito, os AG da dieta, fornecidos como TG, são predominantemente incorporados aos quilomícrons, enquanto os AG fornecidos como PL, parecem ser predominantemente incorporados em partículas de densidade muito baixa sintetizadas nos enterócitos. ^15 , 16^

Além disso, as posições de ligação dos AGPI ao PL ou TG da dieta mostraram efeitos diferenciais na sua absorção e metabolismo. ^1 , 15 , 16^ Por exemplo, a absorção de DHA pode ser aumentada se estiver presente na posição sn-1 do PL da dieta, uma vez que escaparia da hidrólise pela fosfolipase A2 pancreática que libera FA da posição sn-2 do glicerol. ^15^ Além disso, a posição de ligação do EPA e do DHA ao TG também afeta o metabolismo do colesterol e a produção de eicosanoides. ^17^ O EPA ligado à posição sn-1 do TG aumentou a proporção de PGI2 para TXA2 em um grau mais elevado do que o EPA ligado à posição sn-2. É importante lembrar que PGI2 a TXA2 são eicosanoides derivados de AGPI n-6 e têm efeitos opostos.

Portanto, estudos ou recomendações sobre ingestão alimentar ou suplementação de AGPI ômega-3 e ômega-6 devem considerar não apenas ingestão de proporção equilibrada de ômega-6/ômega-3, mas também a qualidade da fonte alimentar e a biodisponibilidade de AGPI dessas fontes dietéticas.
