# Supplementary material for: Papel do Ômega-3 e do Ômega-6 sobre Fatores de Risco Cardiovasculares: Importância da Fonte da Dieta e da Estrutura do Lipídio
Source: Arq Bras Cardiol. 2023 Dec 7;120(11):e20230753. [Article in Portuguese] doi: 10.36660/abc.20230753 (PMC10773457; doi:10.36660/abc.20230753)
Supplement: Translation

# Translation: Role of Omega-3 and Omega-6 on Cardiovascular Risk Factors:Importance of Dietary Sources and Lipid Structure

**Authors:** Victória Moralez Soares, Thais Caroline da Silva, Priscila Portugal dos Santos

**Affiliations:** 1 Brazil Universidade Estadual Paulista Júlio de Mesquita Filho – Campus de Botucatu – Faculdade de Medicina de Botucatu, Botucatu, SP – Brazil

**Keywords:** Die, Mediterranean, Dietary Fats, Risk Factors, Fatty Acids, Omega-3, Fatty Acids, Omega-6, Receptors, Eicosanoid

Omega-3 (α-linolenic acid - ALA, C18:3n-3) and omega-6 (linoleic acid - LA, C18:2n-6) are essential polyunsaturated fatty acids (PUFAs) as they cannot be synthesized by humans or other higher animals. ^1^ These PUFAs play an important role in maintaining cardiovascular health, being extensively studied. Mediterranean diet has been repeatedly associated with lower cardiovascular risk, largely due to the abundance of omega-3. ^2^

In the human body, ALA and LA are converted to other forms of PUFAs, important for human health including eicosapentaenoic acid (EPA, C20:5n-3), docosapentaenoic acid (DPA, C22:5n-3), docosahexaenoic acid (DHA, C22:6n-3) from the omega-3 family, and γ-linolenic acid (GLA, 18:3n-6), dihomo-γ-linolenic acid (DGLA, 20:3n-6) and arachidonic acid (AA, C20: 4n-6) from the omega-6 family. ^1^

PUFAs are generally considered to play a beneficial health role. ^1^ However, they can have opposing effects, with omega-3 being associated with reduced cardiovascular risk and omega-6 being associated with cardiovascular risk factors, ^1 , 3^ as shown in the study by Gonçalinho et al. ^4^ The effects of PUFAs on the body and cardiovascular system are due to the generation of bioactive signaling lipids (derived from DGLA, ARA, EPA, DHA) known as eicosanoids. Among the eicosanoids produced are prostaglandins (PGs), thromboxanes (TXs), and leukotrienes (LTs). Omega-3 can also be metabolized to specialized pro-resolving lipid mediators, such as D-series resolvins, protectins, and maresins. ^1 , 3 , 5^

Omega-6-derived eicosanoids are mainly involved in activating pro-inflammatory response, platelet aggregation, thrombosis, and vascular tone, which can lead to acute cardiovascular disease (CVD). ^2 , 5 , 6^ However, some studies about the effects of omega-6 are still controversial, as discussed by Gonçalinho et al. ^4^ Some AA-derived eicosanoids have opposite effects. For example, TXA2 promotes vasoconstriction and platelet aggregation, leading to thrombus formation. PGI2 is a potent vasodilator and inhibits platelet aggregation. Hence, the balance in producing these eicosanoids establishes vascular tone and thrombotic potential. ^5^ Furthermore, DGLA-derived eicosanoids such as PGE1 are reported to have anti-inflammatory properties and inhibit platelet aggregation mainly. ^3^

Additionally, AA-derived metabolites may also have roles in the resolution of inflammation. For example, lipoxin A4 is a potent pro-resolving mediator, ^7^ and the generation of AA-derived eicosanoids early in the inflammatory response is linked with the later induction of resolution. ^6^ These considerations may be associated with the controversial results found in studies with omega-6.

On the other hand, eicosanoids derived from omega-3 have anti-inflammatory, vasodilatory, and platelet anti-aggregation actions, thus reducing the risk of CVD, ^1 , 2 , 5 , 8^ as shown by Gonçalinho et al. ^4^ Regarding anti-inflammatory effects, EPA and DHA decrease the production of AA-derived eicosanoids, in addition to decreasing activation of the pro-inflammatory transcription factor to decrease the production of inflammatory cytokines and chemokines, acute-phase proteins, and adhesion molecules. ^5^ Additionally, EPA and DHA have platelet anti-aggregation effects to modify eicosanoid profiles. ^9^

Other interesting actions of omega-3 are to regulate lipid and adipocyte metabolism and cholesterol levels, thus enhancing its effect on cardiovascular health. ^1 , 10 , 11^ EPA and DHA beneficially modify blood lipids and have hypotriglycemic effects through the regulation of pathways of lipid synthesis and degradation ^5^ . It also acts to suppress cholesterol biosynthesis enzymes. ^10^ Furthermore, omega-3 acts on brown adipose tissue, which helps energy expenditure through its specialized thermogenic function. ^11^

Finally, omega-3 can influence the effect of saturated fatty acids (SFA) and omega-6 on lipid profile. SFA increases plasma levels of triacylglycerol (TG) only when the diet is deficient in omega-3. Furthermore, in subjects with adequate omega-3 index, the SFA diet does not significantly affect LDL particle concentration or LDL cholesterol levels. ^12^

Considering these opposing effects of omega-3 and omega-6, the proportion of these PUFAs play a significant role in regulating body homeostasis. The conversion rate of LA and ALA to downstream metabolites in humans depends on the ingested omega-3 and omega-6 ratio since they compete for the same enzymes in this process, although a preferential affinity to omega-3 exists. ^1 , 8^ The recommended dietary ratio of omega-6/omega-3 for health benefits is 1:1–2:1. However, in typical Western diets, the ratio is 15:1 to 16.7:1. ^1 , 13^

It is also important to pay attention to the quality of the source of these PUFAs, as the structure of dietary lipids and binding positions of fatty acids (FA) in these lipids can influence their digestion, transportation, and consequently, their bioavailability, metabolism, and effects on the heart. ^14 - 17^

In plant and animal-based foods, the majority (˜98%) of omega-3 is found in the form of TG, followed by phospholipids (PLs) and diacylglycerols, cholesterol esters, and fat-soluble vitamin esters. These different forms of lipids have different levels of bioavailability. ^14^ PLs are more bioavailable due to their amphiphilic nature with superior water-dispersibility and greater susceptibility to phospholipases compared to glycerolysis of TG. ^14^ Studies showed that a lower dose of Krill oil (62.8% compared to fish oil) that contains nearly 35% of DHA in the form of PLs was more efficient than fish oil in promoting absorption of EPA and DHA, since PUFAs in fish oil are found in the form of TG. Furthermore, Krill oil caused fewer adverse effects. ^15 , 18^ Another study reported that infants fed PUFA-supplemented formula from egg PL absorbed omega-3 as efficiently as breastfed infants and better than infants fed a formula containing PUFA-TG. ^19^

Regarding the transport of these lipids, dietary fatty acids provided as TG are predominantly incorporated into chylomicrons in the enterocyte. On the contrary, fatty acids provided as PL seem to be predominantly incorporated into very low-density particles synthesized at the enterocytes. ^15 , 16^

Additionally, binding positions of PUFA to PL or TG from the diet have shown significant differential effects on its absorption and metabolism. ^1 , 15 , 16^ For example, the absorption of DHA can be further increased if it is present in the sn-1 position of dietary PL since it would escape hydrolysis by pancreatic phospholipase A2, which releases FA from the sn-2 position of glycerol. ^15^ Furthermore, the binding position of EPA and DHA to TG also affects cholesterol metabolism and eicosanoid productions. ^17^ EPA bound to the sn-1 position of TG increased the ratio of PGI2 to TXA2 to a higher degree than EPA bound to the sn-2 position. ^17^ It is important to remember that PGI2 to TXA2 are eicosanoids derived from n-6 PUFA and have opposite effects.

Therefore, studies or recommendations on food intake or supplementation of omega-3 and omega-6 PUFA must consider not only a balanced ratio of omega-6/omega-3 but also the quality of the dietary source and the bioavailability of PUFA from these dietary sources.
